# Impact of Putative Probiotics on Growth, Behavior, and the Gut Microbiome of Farmed Arctic Char (*Salvelinus alpinus*)

**DOI:** 10.3389/fmicb.2022.912473

**Published:** 2022-07-19

**Authors:** Stephen Knobloch, Sigurlaug Skírnisdóttir, Marianne Dubois, Laetitia Kolypczuk, Françoise Leroi, Alexandra Leeper, Delphine Passerini, Viggó Þ. Marteinsson

**Affiliations:** ^1^Microbiology Research Group, Matís ohf., Reykjavík, Iceland; ^2^ESBS, University of Strasbourg, Strasbourg, France; ^3^EM3B Laboratory, BRM, IFREMER, Nantes, France; ^4^Faculty of Biosciences, Department of Animal and Aquaculture Sciences, Norwegian University of Life Sciences, Ås, Norway; ^5^Faculty of Food Science and Nutrition, University of Iceland, Reykjavik, Iceland

**Keywords:** probiotics, aquaculture, gut microbiome, salmonid, Mycoplasma, growth, behavior

## Abstract

Beneficial bacteria promise to promote the health and productivity of farmed fish species. However, the impact on host physiology is largely strain-dependent, and studies on Arctic char (*Salvelinus alpinus*), a commercially farmed salmonid species, are lacking. In this study, 10 candidate probiotic strains were subjected to *in vitro* assays, small-scale growth trials, and behavioral analysis with juvenile Arctic char to examine the impact of probiotic supplementation on fish growth, behavior and the gut microbiome. Most strains showed high tolerance to gastric juice and fish bile acid, as well as high auto-aggregation activity, which are important probiotic characteristics. However, they neither markedly altered the core gut microbiome, which was dominated by three bacterial species, nor detectably colonized the gut environment after the 4-week probiotic treatment. Despite a lack of long-term colonization, the presence of the bacterial strains showed either beneficial or detrimental effects on the host through growth rate enhancement or reduction, as well as changes in fish motility under confinement. This study offers insights into the effect of bacterial strains on a salmonid host and highlights three strains, *Carnobacterium divergens* V41, *Pediococcus acidilactici* ASG16, and *Lactiplantibacillus plantarum* ISCAR-07436, for future research into growth promotion of salmonid fish through probiotic supplementation.

## Introduction

Probiotics in aquaculture are defined as live or dead microorganisms administered through the feed or rearing environment that confer a health benefit to their host (Merrifield et al., 2010). Over the past decades, probiotics have attracted a growing interest as a promising new technology to enhance the health and productivity of farmed animals. Aquaculture is currently the fastest growing animal protein sector in the world, expected to continue providing a significant share of animal proteins as the global population increases (FAO, 2018). Research on the use of probiotics in aquaculture has demonstrated several benefits to farmed fish through increased disease resistance, higher survival, enhanced growth performance and improved water quality (Gatesoupe, 1999; Balcázar et al., 2006; Ringø et al., 2020). Probiotics can contribute to the sustainable development of the aquaculture sector, including a reduction of antibiotic use, decreased dependence on wild fish stocks for feed production and a reduced environmental footprint (Wang et al., 2008; Hai, 2015; Olmos et al., 2019). Previous studies have shown that increased disease resistance through probiotic supplementation can be achieved through competitive exclusion of pathogens (Vine et al., 2004a,b; Balcázar et al., 2006; Knipe et al., 2021), the production of antimicrobial compounds (Ravi et al., 2007; Muñoz-Atienza et al., 2013; Tan et al., 2016), or by enhancing the host immune response (Irianto and Austin, 2002; Nayak, 2010; Abumourad et al., 2013). This can lead both to reduced mortality and to increased growth due to reduced pathogen-induced stress (Gatesoupe, 1994; Queiroz and Boyd, 1998; Chang and Liu, 2002; Jha et al., 2015). Other mechanisms involved in growth rate enhancement through probiotic treatment include higher nutrient retention and digestibility through microbial enzymes aiding in nutrient breakdown and the microbial production of vitamins, fatty acids and other nutrients (Bagheri et al., 2008; Mohapatra et al., 2012; Dawood et al., 2019; Mohammadian et al., 2019; Ringø et al., 2020; Tarkhani et al., 2020). In addition, higher nutrient retention and enzymatic breakdown of otherwise inaccessible nutrients can lead to a lower feed conversion ratio and the usability of a wider range of protein sources, potentially replacing fish meal with more sustainable plant proteins in fish feeds (Van Doan et al., 2018; Jang et al., 2019; Serra et al., 2019). Apart from benefits toward health and growth, it has recently been shown that probiotics can influence the behavior of fish (Borrelli et al., 2016). Behavioral characteristics and stress coping styles, which are important indicators of animal welfare, have been linked to the brain-gut axis in fish (Ashley, 2007; Rosengren et al., 2018). Modulating behavior through diets and probiotic supplementation could therefore increase the wellbeing of farmed fish under high stocking densities (Banerjee and Ray, 2017; Soares et al., 2019). Despite large efforts in probiotics research to date, only one bacterial probiotic, a strain of the lactic acid bacterium *Pediococcus acidilactici* licensed under the brand name BACTOCELL^®^ (Lallemand, Canada), has been approved for use in aquaculture in the European Union (Fečkaninová et al., 2017; Serra et al., 2019). In addition, it remains unclear how certain probiotic strains modulate the core fish gut microbiota or how they colonize the gut environment.

The aim of the present study was to evaluate the probiotic characteristics of 10 candidate probiotic bacterial strains through the analysis of *in vitro* assays and their *in vivo* activity on the growth, gut microbiome and behavior of Arctic char (*Salvelinus alpinus*), a commercially farmed salmonid species.

## Materials and Methods

### *In vitro* Strain Characterization

#### Culture Conditions

In total, 10 strains were selected for the *in vitro* assays and probiotic feeding trial (Table 1). These included strains previously showing probiotic properties in fish (*Glutamicibacter bergerei* 04–279, *Enterococcus thailandicus* 04–394), isolated from the fish gut environment (*P. acidilactici* ASG16), or assigned to genera with known probiotic candidates and showing probiotic characteristics, such as antimicrobial activity (*Carnobacterium divergens* V41, *Carnobacterium maltaromaticum* SF1944, *Vagococcus fluvialis* CD264, *Lactococcus lactis* SF1945, *Latilactobacillus sakei* SF1583) or fermentative capacity of feed constituents (*Lactiplantibacillus plantarum* ISCAR-07436, *Levilactobacillus brevis* ISCAR-07433). Two successive cultures of each strain were performed in 1 ml of appropriate medium in 2 ml-deep well plates at 26°C for 24 h. The *Lactiplantibacillus, Levilactobacillus*, *Latilactobacillus* and *Pediococcus* strains were grown in MRS broth (De Man, Rogosa, Sharpe, Biokar Diagnostics, Beauvais, France). The *Carnobacterium, Vagococcus, Lactococcus* and *Enterococcus* strains were grown in BHI broth (Brain Heart Infusion, Biokar Diagnostic, Beauvais, France) and *Glutamicibacter* in Zobell broth (4 g/L Tryptone, 1 g/L yeast extract, and 33.3 g/L aquarium salts). All strains were stored at −80°C in 15% (v/v) glycerol.

**Table 1:** List of strains used in the probiotic feeding experiment.

Treatment group	Species	Strain designation	Cultivation media	Origin	References	Strain collection
P1	*Carnobacterium divergens*	V41	BHI	Fish viscera	Pilet et al., 1995; Remenant et al., 2016	A
P2	*Carnobacterium maltaromaticum*	SF1944	BHI	Smoked salmon	Wiernasz et al., 2017	A
P3	*Vagococcus fluvialis*	CD264	BHI	Peeled shrimps	Wiernasz et al., 2017	A
P4	*Lactococcus lactis*	SF1945	BHI	Smoked salmon		A
P5	*Pediococcus acidilactici*	ASG16	MRS	Atlantic salmon gut		B
P6	*Glutamicibacter bergerei*	04–279	Zobell	Algal concentrate	Lauzon et al., 2010	B
P7	*Enterococcus thailandicus*	04–394	BHI	Fish larval rearing water	Lauzon et al., 2010	B
P8	*Lactiplantibacillus plantarum*	ISCAR-07436	MRS	Fermented vegetables		B
P9	*Levilactobacillus brevis*	ISCAR-07433	MRS	Fermented vegetables		B
P10	*Latilactobacillus sakei*	SF1583	MRS	Smoked salmon		A

Strain collection A: Ifremer/Oniris collection, Nantes, France. Strain collection B: Icelandic Strain Collection and Records/Matís, Reykjavík, Iceland.

#### Gastric Juice Resistance

Deep wells with 24 h-cultures of each strain were centrifuged at 2576 g for 15 min. The supernatant was removed and the cells were washed twice with 1 ml of physiological water using a pipetting robot (VIAFLO 96/384, Integra Biosciences, France). Pellets were then resuspended in 1 ml of phosphate buffer saline solution (PBS, 0.2 M at pH 6). In total, 180 μl of the simulated gastric juice (3 mg/ml of pepsin in 5 g/L of NaCl adjusted at pH 2.5) were distributed into each well of a microplate and inoculated with 20 μl of the PBS suspension of strains. The resistance of the strains was evaluated after 0, 3, and 5 h at 26°C using miniaturized enumeration, as previously described by Wiernasz et al. (2017), on YEG medium (10 g/L Yeast Extract supplemented with 10 g/L of glucose). The sensitivity toward gastric juice was assessed according to the decrease in viable cell concentration after 5 h (Δlog = log CFU/ml at t_0h_−log CFU/ml at t_5h_). Experiments were performed in triplicate.

#### Fish Bile Tolerance

Fish bile was collected from European hakes (*Merluccius mercluccius*) and devil anglerfishes (*Lophius budegassa*) during the French oceanographic cruise EVHOE in November 2019.^1^ Bile acid was retrieved from both species by puncturing the gallbladder with sterile needles and syringes. Pooled fish bile was then stored at −20°C until use.

The probiotic strains were cultured in triplicate and incubated at 26°C for 24 h to reach approximately 10^8^ CFU/ml. A 20 μl of each culture was then inoculated into 180 μl of fish bile solutions diluted in culture media (BHI, MRS or Zobell) to reach a final concentration of 20% fish bile. After 24 h of incubation, cell counts were performed with a miniaturized enumeration as described above. Resistance to fish bile was assessed by the difference of viable cell concentrations after growth, with or without fish bile.

#### Auto-Aggregation Assay

Auto-aggregation was measured by sedimentation characteristics of the candidate strains according to the protocol described by Tareb et al. (2013) with some modifications. Probiotic strains were grown in duplicates in 15 ml of appropriate medium for 48 h at 26°C. A 1.5 ml of each culture was then centrifuged for 5 min at 16200 g. The cells were then washed twice using PBS solution (0.2 M, pH 6) and finally resuspended in PBS to reach an initial OD_600nm_ of 0.3 (ODi). Tubes were incubated at 20°C for 24 h. The absorbance of the upper phase was then measured (OD24h). The percentage of auto-aggregation was calculated according to the following formula:


$$\mathrm{Percentage of auto}-\mathrm{aggregation}=100-\left(\mathrm{OD}2{\mathrm{4h}}^{*}\;100/\mathrm{ODi}\right)$$

#### Antimicrobial Activities

Antimicrobial activities of probiotic strains were assessed using a miniaturized spot-on-lawn method as previously described (Begrem et al., 2020). Briefly, 10 μl of 24 h-probiotic strain cultures were spotted onto soft agar plates inoculated with one of 24 target strains involved in fish diseases, human infections or seafood spoilage (Supplementary Table S1). Culture medium, incubation temperature and inoculation type were adapted according to the target strains (Supplementary Table S1). After 24 to 48 h, clear halos indicated growth inhibition. Toxic activity of BHI and MRS media was excluded by spotting media alone on agar plates with target strains.

#### Hemolytic Activity

Probiotic strains were isolated on blood Columbia agar (VWR, France) and incubated at 26°C or 37°C for 72 h. The β-hemolytic *Staphylococcus aureus* (ATCC 25923) was used as positive control. β-hemolysis capacity led to the formation of a clear halo around a colony and partial α-hemolysis led to a greenish-brown halo.

#### Antibiotic Resistance

Antibiotic resistance of probiotic strains was assessed according to the standard method procedure ISO 10932 (2010) for antimicrobial profiles. The minimum inhibitory concentration (MIC) was determined for nine antibiotics as recommended by EFSA (2012a) with the following concentration ranges: ampicillin (0.125–64 μg/ml), chloramphenicol (0.125–64 μg/ml), clindamycin (0.125–64 μg/ml), erythromycin (0.125–64 μg/ml), gentamicin (1–512 μg/ml), kanamycin (4–2048 μg/ml), streptomycin (4–2048 μg/ml), tetracycline (0.25–128 μg/ml) and vancomycin (0.125–64 μg/ml). The tests were performed in duplicates with an initial concentration of approximately 10^5^ CFU/ml. Microplates were incubated at 26°C for 24 h under aerobic conditions. MICs were determined by measuring the absorbance at 600 nm with a spectrophotometer (Varioskan Lux, Thermo Fisher, France). Strains was considered sensitive when absorbance was inferior to 0.3.

### Feed Preparation and Application of Strains

Basal fish feed was prepared with 9.8% (w/w) fish oil (Laxá, Iceland), 70.3% (w/w) fish meal (Laxá, Iceland), 18.9% (w/w) gelatinized wheat (R2 Agro, Denmark), and 1% (w/w) Farmix mineral and vitamin premix (Trouw Nutrition, The Netherlands). Pellets were prepared as described in Leeper et al. (in press)^2^. In brief, all ingredients were mixed in a standard food mixer (KitchenAid, United States) with a small volume of water and pelleted using a FL82 meat grinder (ADE, Germany). Pelleted feed was then dried in a commercial food dryer (Kreuzmayr, Austria) to a moisture content of less than 10%. The pellet size was approximately 0.5 mm. Protein, fat, water and ash contents of the feed were measured at the analytical service laboratory at Matís, Iceland and were 51.1, 17.6, 6.5, and 10.3%, respectively.

For probiotic supplementation of the diet, each probiotic strain was grown in 50 ml of appropriate liquid culture media on a shaking plate until the late exponential phase. Feed pellets were coated with the strains using sterile spray bottles to achieve a final cell count of 10^6^ CFU g^−1^ feed. The coated pellets were subsequently air dried in a laminar flow hood for 2 h under regular mixing and stored for no more than 2 weeks at 4°C.

### Experimental Set-up

Arctic char fry with approximately 1 g body weight were purchased from a commercial hatchery and transferred to a 1 m^3^ holding tank with a continuous freshwater supply. After an acclimation period of 3 weeks, fish were randomly selected from the holding tank and distributed into 23 circular tanks with 46 fish per tank in total. Water was supplied to the tanks at a rate of 1.75 L min^−1^, exchanging the tank volume of 15 L approximately every 9 min and creating a circular water movement in the tank. Water temperature was continuously logged with iButton DS1922L temperature loggers (Maxim Integrated, United States) and remained between 7.8 and 9.4°C throughout the experiment. Dissolved oxygen remained above 99% and was measured regularly using an oxygen probe (Oxyguard, Denmark). After an additional acclimatizing period of 5 days, the feeding trial was started. Each tank was supplied with either one of the 10 probiotic diets (no replicates were conducted due to a limited number of tanks available) or a control diet (in triplicate) for 4 weeks, after which all tanks received the control diet for the remaining 4 weeks (for a timeline of the experiment see Supplementary Figure S1). The feed was supplied to the tanks six times per day using a conveyor belt set to feeding 6.5% of the fish body weight per day. This value was higher than commercial feeding recommendations in order to ensure maximum uptake of feed over the trial period. Tanks were cleaned regularly to prevent excessive biofilm growth on the tank walls. The experiment was performed according to European and Icelandic guidelines under the license FE-1134 from the Icelandic Food and Veterinarian Authority and UST201707 from the Icelandic Environment Agency.

### Growth Assessment and Sample Collection

All fish were batch weighed at the start of the feeding trial, at week 4 and at the end of the trial at week 8. The fish were fasted for 12 h prior to weighing and sample collection. Before weighing, the fish were anesthetized with 350 ppm of phenoxyethanol, gently dried with paper tissue and weighed on a precision scale (Shinko Denshi, Japan). The specific growth rate (SGR) was calculated with the following formula:


$$\mathrm{SGR}={\left[\ln\left(\mathrm{final weight}\right)-\ln\left(\mathrm{initial weight}\right)\right]}^{*}\;100/\mathrm{days}$$

For gut histology and metataxonomic analysis, five fish per tank were randomly selected at week 4 and week 8, and euthanized with 500 ppm of phenoxyethanol. Fish were then transported on ice to the laboratory, rinsed first with ethanol and then with sterile laboratory grade water to remove transient bacteria from the skin. The fish were dissected and the mid and hindgut was removed, cut into approximately 1 mm wide segments and frozen at −80°C until DNA extraction. One 1 mm segment from each hindgut was fixed in freshly prepared 4% paraformaldehyde in 1 × PBS for 24 h at 4°C and then transferred to 70% ethanol at 4°C for long-term storage prior to histology.

### Histology and FISH

Gut histology and 16S rRNA fluorescence *in situ* hybridization (FISH) were performed for fish in treatment groups P1 and P8 after week 4 and week 8 to determine the location of viable probiotic cells in the digesta and mucosa of the hindgut. These two groups were selected due to their increased growth rate compared to the control group. Fixed gut segments were dehydrated in successive baths of 80, 90, 95, and 100% ethanol, followed by a bath in xylene and embedding in paraffin. Paraffin embedded gut segments were cut into 5 μm sections on a CM1800 microtome (Leica, Germany) with MX35 Ultra microtome blades (Thermo scientific, United States) and deparaffinized with xylene followed by a wash in 100% ethanol. Histological sections were hybridized with a mix of Cy3 labeled probes CDV175 and CDV462 (treatment P1) or LBP457 (treatment P8), as well as Alexa488 labeled universal bacterial probe EUB338 (Supplementary Table S2). Hybridization and epifluorescence microscopy was performed as previously described in Knobloch et al. (2019) with the exception of using 40% formamide in the hybridization buffer. Epifluorescence images were processed in daime v. 2.2 (Daims et al., 2006).

### Gut Metataxonomic Analysis

#### DNA Extraction, PCR, and Sequencing

DNA was extracted from defrosted fish guts as previously described in Leeper et al. (2022) using the QIAamp PowerFecal Pro DNA Kit (Qiagen) with modifications. Two negative extraction control samples containing only zirconia/silica beads and CD1 buffer were included and processed alongside the other samples. All samples were subjected to PCR amplification and Illumina sequencing of the partial 16S rRNA gene as previously described in Knobloch et al. (2021) using the universal prokaryotic primer pair S-D-Bact-0341-b-S-17 (5’-CCTACGGGNGGCWGCAG-3′) /S-D-Bact-0785-a-A-21 (5’-GACTACHVGGGTATCTAATCC-3′; Klindworth et al., 2013) and high-fidelity Q5 polymerase (New England Biolabs, United States).

#### Microbial Community Analysis

Bioinformatic analysis and subsequent statistical analysis were performed in R version 4.0.2 (R Core Team, 2020) implemented in RStudio (RStudio Team, 2016). Amplicon sequence variants (ASVs) were inferred using the R package DADA2 version 1.16.0 (Callahan et al., 2016). In brief, raw reads were truncated after 260 bp (forward read) and 240 bp (reverse read) and primer sequences were removed. After denoising and merging the forward and reverse reads, ASVs within the size range of 410 to 460 bp were retained and assigned a taxonomic rank using the Silva v138 reference database (Quast et al., 2013). ASVs present in either one of the negative controls above 1% of the relative abundance were removed from the dataset. Microbial community analysis was performed in R package phyloseq (McMurdie and Holmes, 2013). Differential abundance analysis was performed with an R-implementation^3^ of ANCOM (Mandal et al., 2015) using the Benjamini–Hochberg procedure and an alpha of 0.05. ASVs were considered significantly more or less abundant at a detection cut-off of 0.7. Plots were created with package ggplot2 (Wickham, 2009).

### Behavioral Analysis

Stress response was analyzed based on time spent moving in a confined space according to Øverli et al. (2006). At time T1, six fish were randomly selected from each tank and placed individually into a 1 L glass beaker filled with 300 ml of water. Each beaker was separated by an opaque screen in order to prevent the fish from observing each other. Overhead lighting was uniform across all beakers. An overhead camera recorded the movement between 0 to 10 min and 20 to 30 min after transfer to the confined space. Time spent moving was calculated using the software Solomon Coder version 19.08.02 (Péter, 2011). Movement was defined as active locomotion transporting the fish further than an estimated 10% of its body length or active swimming against the walls of the tank. Differences between treatments were analyzed by analysis of variance (ANOVA). Dunnett’s comparison was conducted as *post-hoc* test, as well as independently to detect significant differences between individual treatments and the control group according to Hothorn (2016).

## Results

### *In vitro* Characterization of Candidate Probiotic Strains

The probiotic potential of the 10 bacterial strains was evaluated *in vitro*, measuring resistance to fish digestive conditions and auto-aggregation required for intestinal colonization, as well as antimicrobial activities against aquaculture pathogens and other undesirable bacterial species. In addition, safety of the strains was assessed by hemolytic capacity and antibiotic resistance evaluation.

**Figure 1:**
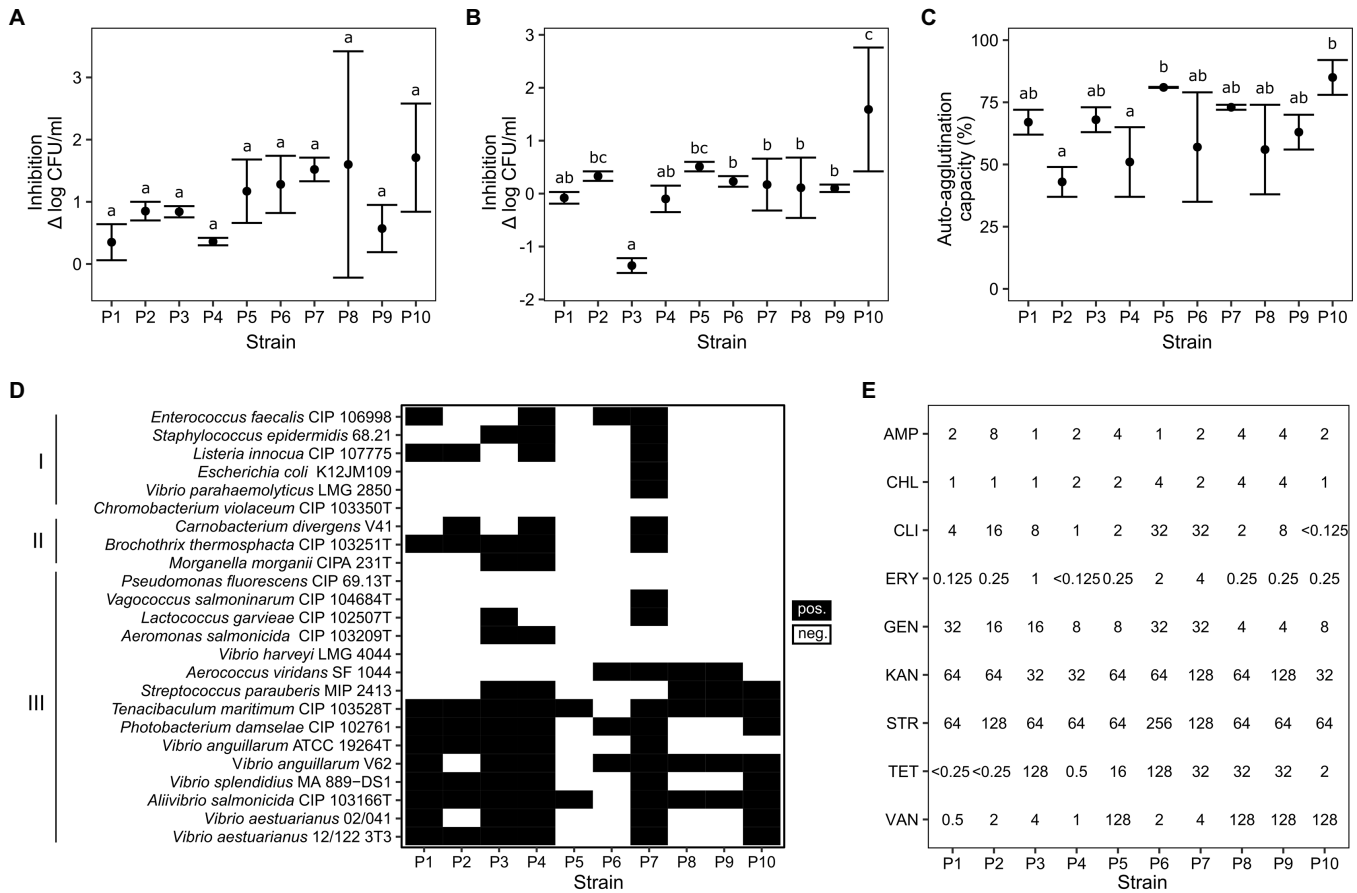
Results of *in vitro* assays. **(A)** Survival in simulated gastric juice after 5 h (ANOVA: *F*(9, 20) = [1.58], *p* > 0.1); **(B)** Survival in bile acid after 24 h (ANOVA: F(9, 20) = [7.51], *p* < 0.001); **(C)** Auto-aggregation capacity (ANOVA: F(9, 20) = [4.37], *p* < 0.005); **(D)** Antimicrobial activity profiles against bacteria associated with human infection (I), seafood spoilage (II), and fish diseases (III). **(E)** Minimal inhibitory concentrations against nine antibiotics in μg/ml (AMP: ampicillin, CHL: chloramphenicol, CLI: clindamycin, ERY: erythromycin, GEN: gentamicin, KAN: kanamycin, STR: streptomycin, TET: tetracycline, VAN: vancomycin). See selected EFSA cut-off values in Supplementary Table S3. P1: *Carnobacterium divergens*; P2: *Carnobacterium maltaromaticum*; P3: *Vagococcus fluvialis*; P4: *Lactococcus lactis*; P5: *Pediococcus acidilactici*; P6: *Glutamicibacter bergerei*; P7: *Enterococcus thailandicus*; P8: *Lactiplantibacillus plantarum*; P9: *Levilactobacillus brevis*; P10: *Latilactobacillus sakei*. Letters above error bars indicate significant differences (*p* < 0.05) between samples based on Tukey’s HSD *post-hoc* test.

All strains displayed high resistance to gastric juice and bile acid, particularly *C. divergens* V41 (P1) and *L. lactis* SF1945 (P4) which showed less than log 0.4 CFU/ml inhibition against gastric juice and no inhibition against bile acid (Figure 1). Interestingly, *V. fluvialis* CD264 (P3) showed increased growth with fish bile compared to the control. The percentage of auto-aggregation ranged from 43 to 85%, with the highest values for *Lactobacillus sakei* SF1583 (P10) and *P. acidilactici* ASG16 (P3) with 85 ± 7% and 81 ± 0.2%, respectively. A comparison between strains showed no significant difference for gastric juice resistance (ANOVA: *F*(9, 20) = [1.58], *p* > 0.1), but significant differences for fish bile resistance (ANOVA: F(9, 20) = [7.51], *p* < 0.001) and auto-aggregation (ANOVA: F(9, 20) = [4.37], *p* < 0.005; Figure 1).

Antimicrobial activity profiles were strain-dependent. Except *P. acidilactici* ASG16 and *Arthrobacter bergerei* 04–279, all LAB strains showed antimicrobial properties against the tested aquaculture pathogens (Figure 1). The *Carnobacterium* strains (P1, P2), *L. lactis* SF1945 (P4) and *E. thailandicus* 04–394 (P7) were also able to inhibit seafood spoilage organisms and *Listeria* sp.

No definite hemolytic activities were detected except for light green halos for *V. fluvialis* CD264 (P3) and *L. sakei* SF1583 (P10) which could possibly be related to H_2_O_2_ production (data not shown). Due to a lack of reported threshold values it was not possible to establish a resistance or sensitivity phenotype with high confidence for the tested strains. However, the resistance of the *Lactiplantibacillus* sp., *L. lactis* and the *Pediococcus* sp. can be assessed by comparison to EFSA guidelines available only for these feed additives species (Supplementary Table S3; EFSA, 2012a). The strain *L. sakei* SF1583 (P10) was found susceptible to all nine antibiotics. The obligate heterofermentative *L. brevis* (P9) seemed to display kanamycin and clindamycin resistances with MICs of 128 μg/ml and 8 μg/ml respectively, much higher than the EFSA cut-off values of 32 μg/ml and 1 μg/ml. The other strains showed intermediate resistance to streptomycin for *L. lactis* SF1945 (P4), tetracycline for *P. acidilactici* ASG16 (P5) and ampicillin for *L. plantarum* ISCAR-07436 (P8), with MIC values close to the reported cut-off values.

### Fish Growth and Survival

**Figure 2:**
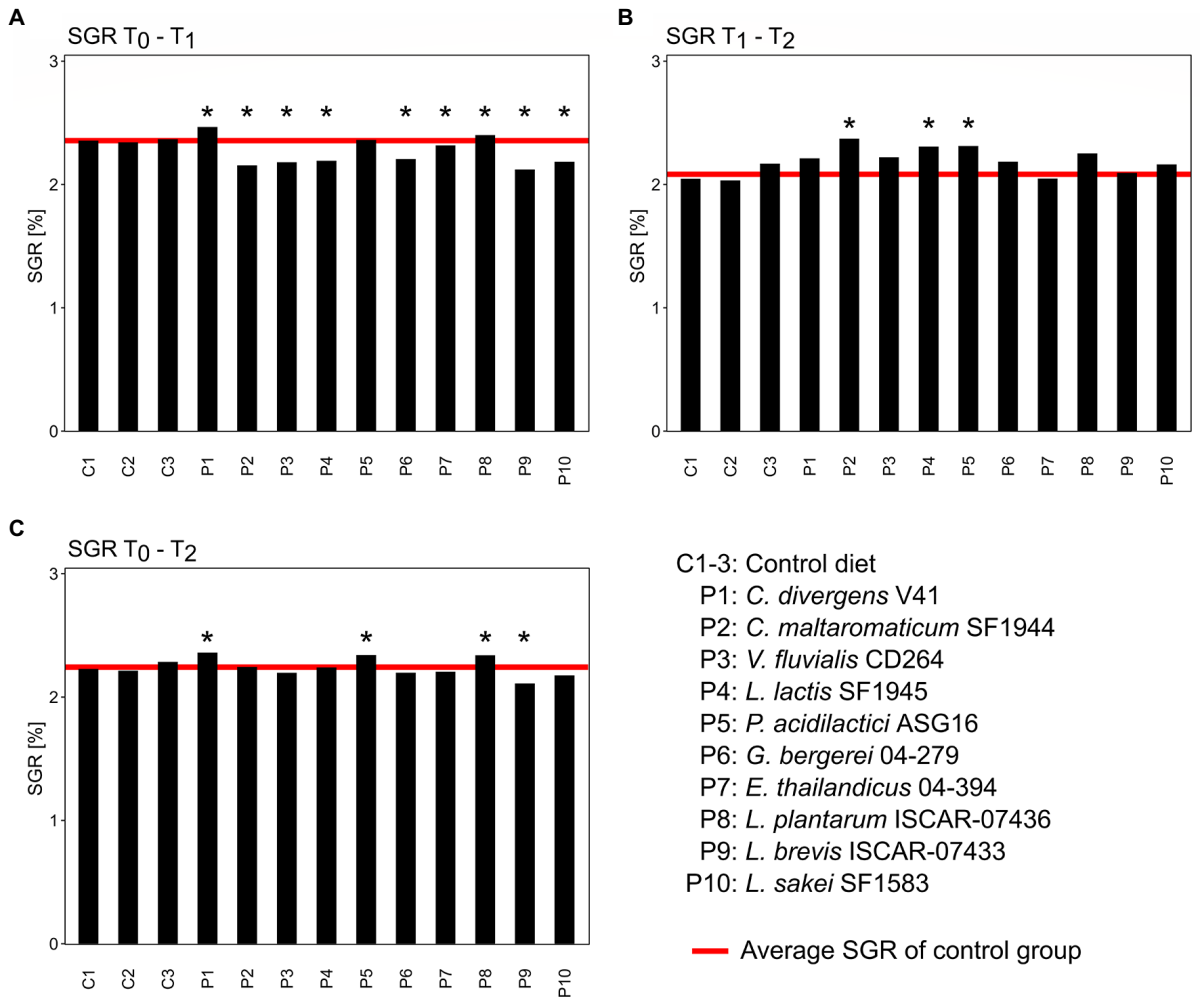
Specific growth rates (SGR) of the different treatment groups (P1–P10) compared to the control (C1–C3) between the first 4-week growth period **(A)**, the second 4-week growth period **(B)** and the entire 8-week growth period **(C)**. Asterisks above bars indicate significant difference of the control groups to the treatment group based on one-sample *t*-tests (*p* < 0.05).

The average SGR of Arctic char fry fed with the control diet was 2.36 ± 0.01% during the first 4 weeks of the trial (Figure 2). Only the group P1, supplemented with *C. divergens* V41, and P8, supplemented with *L. plantarum* ISCAR-07436, had a higher SGR of 2.47 and 2.40%, respectively. One-sample t-tests showed that the control group was either significantly higher or lower (*p* < 0.05) than each of the treatment groups apart from group P5, supplemented with *P. acidilactici* ASG16. Between week 4 and 8 of the growth trial, the average SGR of the control group was 2.08 ± 0.06%. All groups, apart from P9, previously fed with *L. brevis* ISCAR-07433, had a higher growth rate than the control group during this period. However, the control only significantly differed from treatment groups P2, P4 and P5. Across the entire 8-week growth period only groups P1, P5 and P8, with an SGR of with 2.36, 2.34 and 2.34%, respectively, had a higher SGR than the control group, which was significantly lower with an SGR of 2.24 ± 0.03%. The control group was only significantly higher than treatment group P9, which showed the slowest growth performance over the whole growth trial. No mortality occurred due to the treatments throughout the 8-week experiment.

### Gut Microbiota

**Figure 3:**
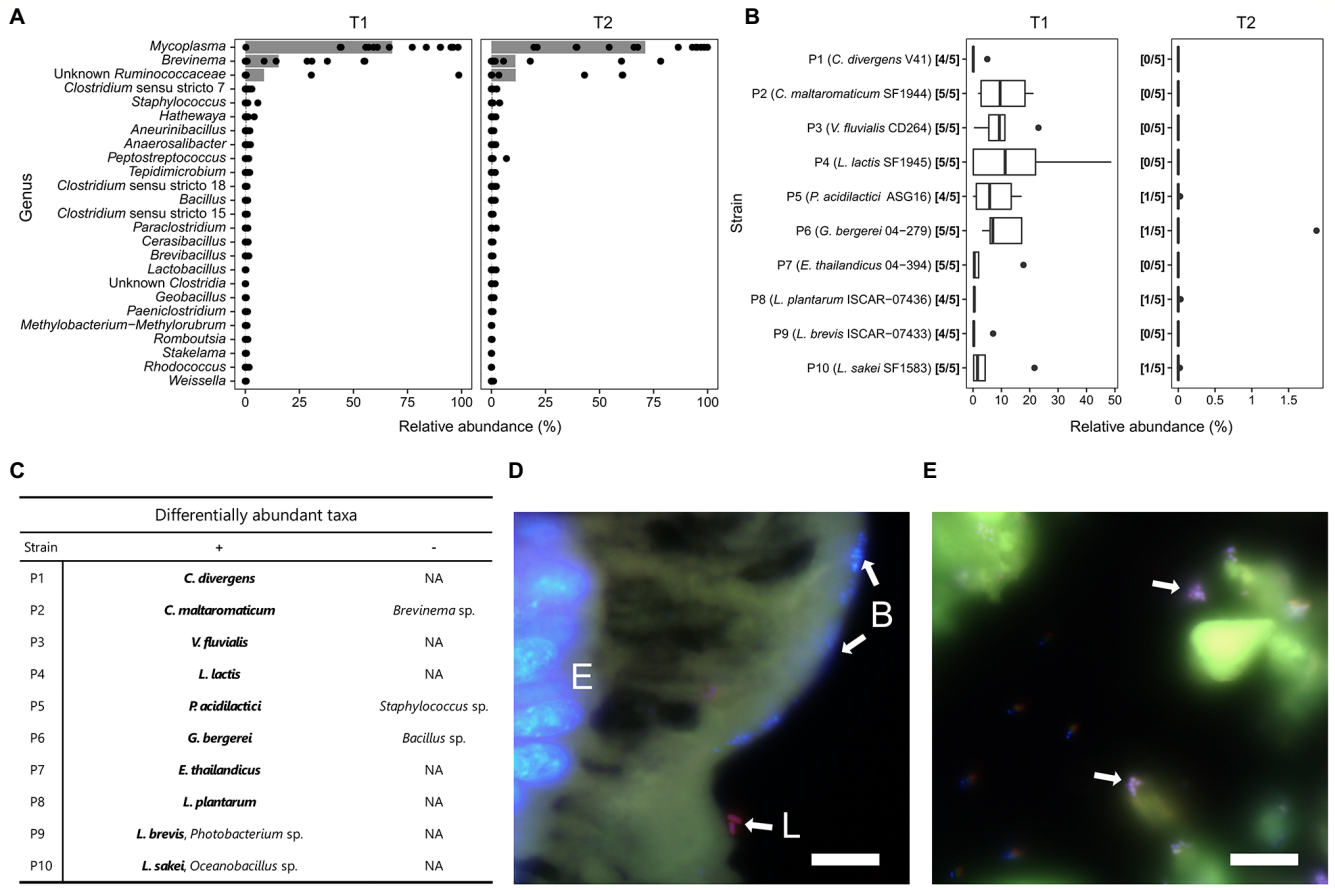
Description of Arctic char gut microbiota. **(A)** Relative abundance of genera (or higher taxon if genus is unknown) in fish guts of the control group (*n* = 15) at time T1 and T2. Gray bars indicate average relative abundance; **(B)** Relative abundance of probiotic strains detected in fish guts of the treatment groups (*n* = 5) at time T1 and T2. Positive detection was assumed if the 16S rRNA gene sequence matched the reference 16S rRNA gene of the respective probiotic strains with 100% sequence similarity; **(C)** Differentially abundant taxa for each treatment group with significantly higher (+) or lower (−) relative abundance compared to the control group. Probiotic strains are marked in bold. **(D)** FISH image of gut epithelium of treatment group P8 with nuclei of enterocytes **(E)** and other bacteria **(B)** stained blue (DAPI), and *L. plantarum* cells (L.) stained purple (DAPI + Cy3 + Alexa488). Bar: 10 μm; **(E)** FISH image of intestinal lumen of treatment group P1 with *C. divergens* cells marked purple (DAPI + Cy3 + Alexa488). Bar: 10 μm.

The gut microbiota of fish in the control group was dominated by an ASV assigned to the genus *Mycoplasma*, accounting for over 67% of the average relative abundance at time T1 and T2 (Figure 3A). The second and third most abundant ASVs were assigned to the genus *Brevinema* and to the family *Ruminococcaceae*, respectively. Although the relative abundance of both taxa varied strongly between samples, together they accounted for an average of 23% of the relative abundance in the control group across times T1 and T2. Each of the other taxa detected contributed less than 1% of the average relative abundance of the fish gut microbiota and were not present in all examined individuals.

In the groups receiving feed supplemented with the probiotic strains, the percentage of each respective strain detected in the gut varied strongly by the individual examined and by the strain applied (Figure 3B). At time T1, the highest average relative abundances were found for P4, P2, P6, P3 and P5 with 16.4, 10.7, 10.1, 9.9 and 7.5%, respectively. In addition, the strains were not detected in all fish fed with the probiotics. One out of five individuals fed with either P1, P5, P8 and P9 did not harbor detectable levels of the corresponding strain at the time of sampling. At time T2, after being fed the control feed without probiotics for 4 weeks, only four strains were detected in one out of five individuals per treatment with 0.02 to 1.9% of the relative abundance (Figure 3B).

Although present in the fish gut until T1, the strains did not have a large impact on the abundance of the three dominant taxa found in the gut microbiome (Figure 3C). Only the group fed with P2 showed a significant reduction of *Brevinema* compared to the control group. Fish fed with P5 and P6 showed a significant reduction of the minor taxa *Staphylococcus* and *Bacillus*, respectively. Those fed with P9 and P10 harbored a higher proportion of *Photobacterium* and *Oceanobacillus*, respectively, compared to the control group.

FISH imaging of hindgut sections from fish supplemented with *C. divergens* V41 and *L. plantarum* ISCAR-07436 showed that intact cells were found exclusively outside of the mucus, compared to dense clusters of bacterial cells, likely belonging to the dominant *Mycoplasma* sp., found within the mucus layer of the fish gut epithelium (Figures 3D,E). No cells marked with *C. divergens* or *L. plantarum* specific probes were detected in fish gut section from time T2.

### Behavioral Analysis

**Figure 4:**
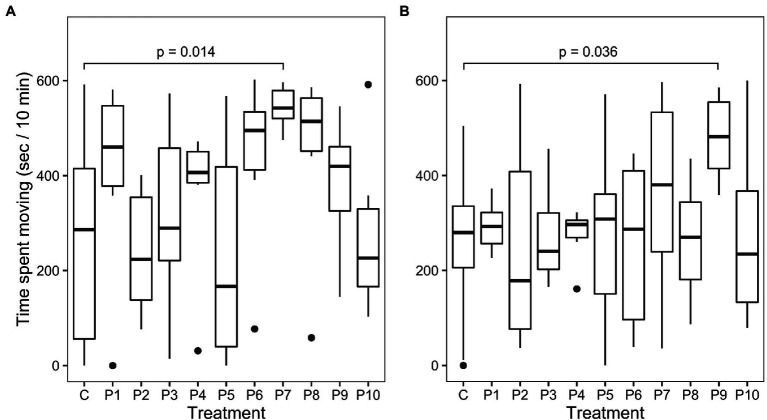
Time spent moving of control **(C)** and treatment groups (P1–P10) during confinement. **(A)** 0–10 min after transfer to confinement. **(B)** 20–30 min after transfer to confinement.

Behavior analysis was based on locomotive activity in a confined space by individuals in the control group (*n* = 18) compared to individuals in each of the other treatment groups (*n* = 6 per group). There was a significant effect of treatment in the first observation period based on ANOVA (*F*_(10, 67)_ = 2.05, *p* = 0.04). Dunnett’s test indicated that the mean time spent moving was significantly different (Figure 4, *p* = 0.014) between the control group (255.3 ± 198.4 s) and the treatment group P7 receiving *E. thailandicus* 04–394 (543.6 ± 45.8 s). Average time spent moving was more similar between the control and treatment groups during the second observation period without significant differences between treatments based on ANOVA. However, Dunnett’s test showed a significant difference (*p* = 0.036) of the time spent moving between the control group (260.9 ± 140.9 s) and treatment group P9, previously fed with *L. brevis* ISCAR-07433 (479.9 ± 92.2 s).

## Discussion

*In vitro* assays can be useful for pre-selection of strains with putative probiotic properties *in vivo* (Vine et al., 2004a; Guo et al., 2010; Vinderola et al., 2017). In this study, no strain showed complete inhibition by gastric juice or bile acid indicating their survival in the fish gastrointestinal system. Strains *C. divergens* V41 and *L. lactis* SF1945 displayed the highest resistance in both tests making them highly suitable probiotic candidates in this regard. Average auto-aggregation capacity varied but remained above 40% for all strains. Previous studies on auto-aggregation of human and animal probiotic candidates showed similar values (Tareb et al., 2013; Krausova et al., 2019), indicating that all candidate strains could have the potential to adhere to epithelial cells, particularly *Latilactobacillus sakei* SF1583 and *P. acidilactici* ASG16 which displayed the highest values. However, as reported in Ouwehand and Salminen (2009), *in vivo* assays are needed to validate this hypothesis. Antimicrobial activity against specific pathogens and spoilage organisms was present in all strains, showing potential beneficial characteristics for application in aquaculture (Pan et al., 2008; Muñoz-Atienza et al., 2013; Yi et al., 2018). Antibiotic assays and hemolytic activities provided safety assessment of strains. These results can be useful for the Qualified Presumption of Safety (QPS) status of LAB deliberately introduced into the feed chain. No clear hemolytic activities were observed. However, the results seemed to show some antibiotic resistances. Among the LAB species with known cut-off values (EFSA, 2012a), *L. brevis* ISCAR-07433 is the only strain that probably harbors antibiotic resistance to clindamycin and kanamycin. Other putative resistances were observed for *L. lactis* SF1945, *P. acidilactici* ASG16 and *L. plantarum* ISCAR-07436. For strains without existing threshold values, the MICs of a large number of isolates should help to determine the cut-off values. Genome sequencing and analysis would be required to identify resistance genes and their transferability by mobile genetic elements and to further assess the risks of conferring resistance to fish and humans.

The SGR reported in this study is in good accordance with previous studies on growth rates of juvenile Arctic char for similar age groups (Jobling et al., 1993; Yang and Dick, 1994; Gunnarsson et al., 2011). Supplementation of probiotic strains impacted growth both throughout the treatment period (week 1 to 4) as well as after supplementation had ended (week 5 to 8). Fish fed with *C. divergens* V41 and with *L. plantarum* ISCAR-07436 showed an increased growth rate during the 4-week treatment period which led to a slightly increased growth rate over the total 8-week growth trial compared to the control group. Probiotics have previously been shown to increase the growth rate of farmed fish species as, for instance, in juvenile *Labeo rohita* fed with a combination of three probiotics (*Bacillus subtilis*, *L. lactis* and *Saccharomyces cerevisiae*; Mohapatra et al., 2012), in *Oreochromis niloticus* fed with the yeast *S. cerevisiae* (Lara-Flores et al., 2003), or in *Paralichythys olivaceus* fed with a strain of *L. lactis* (Nguyen et al., 2018). According to these studies, the impact on growth could be the result of microbial metabolites or the enzymatic digestion of feed constituents, chiefly proteins, aided by live probiotics. This, in turn, could lead to higher nutrient digestion and utilization by the host. The study by Nguyen et al. (2018) compared the gut metabolome of fish fed with and without probiotics showing that the gut environment of the probiotic-fed group was significantly enriched in metabolites such as citrulline, tricarboxylic acid cycle intermediates, short chain fatty acids, vitamins and taurine which could aid in host nutrition. Both *Carnobacterium* spp. and *Lactiplantibacillus* spp. have previously been isolated from the gut of different fish species and have shown proteolytic and lipolytic activity *in vitro* (Sahnouni et al., 2016). Hence the observed growth rate increase detected in this study could be the result of increased nutrient availability. The indication that the weight increase was sustained even after probiotic supplementation was terminated shows that even short-term application of these strains could promote a growth advantage during the juvenile development stage. The group fed with *P. acidilactici* ASG16 did not show an increased growth rate until the second half of the trial when the probiotic treatment was not applied anymore, indicating that the strain did not actively increase nutrient availability to the host. *P. acidilactici* is one of the most widely studied probiotic bacteria for fish and attributes of *P. acidilactici* supplementation include the reduction of bone deformation in developing fish (EFSA, 2012b), increased growth performance (Ashouri et al., 2018; Arani et al., 2020), increased stress resistance (Taridashti et al., 2017) and enhanced disease resistance or mucosal immune response (Rahimnejad et al., 2018; Hoseinifar et al., 2019). Whereas it is likely that no active nutritional benefit was gained through *P. acidilactici* in this study, it is possible that other beneficial properties of the strain conferred a health benefit in the host resulting in increased growth in the long term.

The other seven strains tested in this study did not promote growth benefits and even appeared to reduced growth during the treatment period. Most studies published on the effect of putative probiotic in farmed fish report positive correlations between probiotic supplementation and growth rates (Ringø et al., 2020). However, Hoseinifar et al. (2019) and Batista et al. (2016) showed significantly decreased growth rates in fish fed with a *P. acidilactici* strain and a multispecies probiotic, respectively, and several studies have shown a correlation between differences in the gut microbial composition and reduced growth (Chapagain et al., 2019; Jin et al., 2021). The reasons for decreased growth in this study are not clear. Four of the seven strains that induced slower growth rates in the fish were also detected at higher relative abundances in the gut, suggesting rapid growth of the strains *in vivo*. This could have led to, for instance, competition for nutrients otherwise available to the host, changes of the pH in the gut environment impairing nutrient absorption, crowding out of resident bacteria or causing local inflammation and other host immune reactions. Despite low growth while being fed the bacterial strains, six of the groups could regain their weight relative to the control group during the second period of the trial through compensatory growth. This phenomenon has previously been detected in Arctic char transferred to warm water after a period in suboptimal rearing in cold water (Mortensen and Damsgård, 1993). Only the group fed with *L. brevis* ISCAR-07433 showed a continuously decreased growth rate throughout the experiment, albeit without any cases of mortality. Future studies might focus on the cause of this interaction to elucidate bacterial characteristic to avoid when screening for growth enhancing probiotic strains for salmonid fish.

The gut microbiota of the juvenile Arctic char was dominated by three bacterial taxa, a *Mycoplasma* sp., *Brevinema* sp. and an unknown *Ruminococcaceae*. *Mycoplasma* has previously been described as a gut commensal across different wild and farmed salmonid species with potential mutualistic properties (Llewellyn et al., 2015; Mora-Sánchez et al., 2020; Rasmussen et al., 2021), suggesting an important association with its host in the present study. The sole cultivated member of the genus *Brevinema* is *B. andersonii,* an infectious spirochete (Defosse et al., 1995). However, less than 91% sequence similarity to this type species indicates that the bacterium detected in the Arctic char guts could belong to a separate genus. A sequence comparison to the NCBI nucleotide collection showed highest sequence similarity of the partial 16S rRNA gene to an uncultured bacterium detected in the Long-Jawed Mudsucker, *Gillichthys mirabilis* (96.3% sequence similarity), followed by a strain detected in the herbivorous marine fish *Naso tonganus* (96.1%), further highlighting its association with the gut of fish species. Likewise, the unknown *Ruminococcaceae* showed highest sequence similarity to an isolate from a fish species (*Siganus canaliculatus*) with 90.3% sequence similarity. The low abundance of the other taxa detected in the gut suggests that many are transient microorganisms and not members of the autochthonous gut microbiota. This is the first study characterizing the gut microbiota of farmed Arctic char. Compared to previous studies on wild Arctic char, the farmed fish appear to harbor a lower number of permanent members of the gut microbiota, but partially share the presence and high relative abundance of *Mycoplasma* and *Brevinema* (Hamilton et al., 2019; Element et al., 2020, 2021).

Probiotic treatment did not affect the relative abundance of the three dominant taxa in this study, with the exception of *C. maltaromaticum* SF1944 which was associated with a significantly reduced relative abundance of the *Brevinema* sp., possibly due to competition for the same niche. *Brevinema*, as well as *Staphylococcus* and *Bacillus*, the other two taxa with a significant reduction in abundance after probiotic treatment, have all been previously detected in farmed fish gut microbiomes, sometimes representing over a quarter of the relative community abundance (Parata et al., 2020; Skrodenytė-Arbačiauskienė et al., 2021; Leeper et al., 2022) and therefore likely benign to the host organism.

Overall, these results highlight the stability of the autochthonous gut microbiota toward perturbation by exogenous microorganisms. This is in contrast to previous studies which have shown that probiotic bacteria modulate the fish gut microbiome (Geraylou et al., 2013; Ramos et al., 2013; Lobo et al., 2014). This difference might be due the limited number of core gut microorganisms found in the juvenile Arctic char and therefore a lack of metabolic or functional redundancy which, in turn, necessitates a stable microbial community. This might also explain a lack of long-term survival of the strains in the gut as there may not have been a suitable niche for colonization. However, the presence of intact cells of *C. divergens* and *L. plantarum* in the gut, detected through FISH, indicates survival of these strains in the gut environment and the possibility of active metabolism. *C. divergens* has previously been detected in the gut of Arctic char (Ringø et al., 2006) which further points toward a functional, possibly mutualistic, role in the gut.

As in other animals, there is mounting evidence that the gut microbiome can modulate the behavior of fish *via* the gut-brain-axis and that probiotics can influence this interaction (Borrelli et al., 2016; Davis et al., 2016a,b). The current study shows first evidence of bacterial strains altering the stress response in a farmed salmonid. Whereas most strains did not show a significant difference in motility under confinement stress, fish fed with *E. thailandicus* 04–394 and *L. brevis* ISCAR-07433 showed significantly increased motility compared to the control group and thereby lower stress coping ability. It is not clear what mechanism triggers this response, but both treatments were also associated with slower growth during the treatment period and for *L. brevis* ISCAR-07433 even sustained slow growth after the treatment period had ended. Gut inflammation was not analyzed in this study, however, previous studies on mouse models have shown a correlation between inflammation and behavioral changes (Bercik et al., 2010; Guida et al., 2018). Hence, the *E. thailandicus* and *L. brevis* strains could have caused local immune responses in the gut which, in turn, could have led to inflammation, reduced nutrient absorption and behavioral changes.

## Conclusion

Screening 10 bacterial strains for probiotic properties demonstrated both beneficial and adverse effects on host growth and behavior in juvenile Arctic char. *In vitro* assays can aid in the pre-selection of probiotic strains based on strain characteristics and safety status, however, there was no clear correlation between *in vitro* results and *in vivo* performances in this study. Based on small-scale growth trials, three putative probiotics, *C. divergens* V41, *L. plantarum* ISCAR-07436 and *P. acidilactici* ASG16, were highlighted as promising strains with the ability to enhance growth performance in juvenile Arctic char without altering behavioral characteristics or the gut microbiota. Moreover, *C. divergens* and *L. plantarum* are on the QPS list, with some strains currently being commercialized in food applications. Further studies are needed to analyze the benefits of these probiotic treatments during different growth stages and in different salmonid species, as well as their impact on performance throughout the entire rearing period. In-depth analysis of the metabolic potential of these strains *in vivo* will elucidate the beneficial interactions within the host organism and the underlying mechanisms of growth enhancement.

## Data Availability Statement

Raw 16S rRNA gene sequences are deposited in the NCBI Sequence Read Archive under BioProject PRJNA791800.

## Ethics Statement

The animal study was reviewed and approved by The Icelandic Food and Veterinarian Authority under licence FE-1134 and UST201707 from the Icelandic Environment Agency.

## Author Contributions

SK, SS, LK, FL, DP, and VM contributed to the conception and design of the study. SK, SS, MD, and AL conducted field and laboratory work. AL provided expert advice on feed production and feeding trials. SK wrote the first draft of the manuscript. LK and DP wrote sections of the manuscript. All authors contributed to the article and approved the submitted version.

## Funding

This study was supported by the EU H2020 Research and Innovation program (grant no. 818368) and the Icelandic AVS fund (grant R 17 018-17).

## Conflict of Interest

The authors declare that the research was conducted in the absence of any commercial or financial relationships that could be construed as a potential conflict of interest.

## Publisher’s Note

All claims expressed in this article are solely those of the authors and do not necessarily represent those of their affiliated organizations, or those of the publisher, the editors and the reviewers. Any product that may be evaluated in this article, or claim that may be made by its manufacturer, is not guaranteed or endorsed by the publisher.

## Footnotes

^1^ http://dx.doi.org/10.17600/18000878

^2^Leeper, A., Benhaïm, D., Smárason, B., Knobloch, S., Ómarsson, K., Bonnafoux, T., et al. (in press). Feeding black soldier Fly larvae (Hermetia illucens) reared on food waste alters gut characteristics of Atlantic salmon (Salmo salar). J. Insects as Food Feed.

^3^ https://github.com/FrederickHuangLin/ANCOM
