# Supplementary material for: Impact of Putative Probiotics on Growth, Behavior, and the Gut Microbiome of Farmed Arctic Char (Salvelinus alpinus)
Source: Front Microbiol. 2022 Jul 19;13:912473. doi: 10.3389/fmicb.2022.912473 (PMC9343752; doi:10.3389/fmicb.2022.912473)
Supplement: Supplementary file 1 [file Table_1.DOCX]

**Supplementary Figure S1.** Schematic overview of the growth experiment.
